# A previously unreported potential malaria vector in a dry ecology of Kenya

**DOI:** 10.1186/s13071-019-3332-z

**Published:** 2019-02-11

**Authors:** Edwin O. Ogola, Edith Chepkorir, Rosemary Sang, David P. Tchouassi

**Affiliations:** International Centre of Insect Physiology and Ecology (icipe), P.O. Box 30772-00100, Nairobi, Kenya

**Keywords:** *Anopheles funestus* group, Malaria transmission, Entomological surveillance, Molecular approaches, Dry ecology, Kenya

## Abstract

**Background:**

In Kenya, malaria remains a major public health menace equally affecting the semi-arid to arid ecologies. However, entomologic knowledge of malaria vectors in such areas remains poor.

**Methods:**

Morphologically-identified wild-caught *Anopheles funestus* (*s.l.*) specimens trapped outdoors from the semi-arid to arid area of Kacheliba, West Pokot County, Kenya, were analysed by PCR and sequencing for species identification, malaria parasite infection and host blood-meal sources.

**Results:**

Three hundred and thirty specimens were analysed to identify sibling species of the *An. funestus* group, none of which amplified using the available primers; two were infected with *Plasmodium falciparum* and *Plasmodium ovale*, separately, while 84% (*n* = 25) of the blood-fed specimens had fed on humans. Mitochondrial cytochrome *c* oxidase subunit 1 (*cox*1) and nuclear ribosomal internal transcribed spacer 2 (ITS2) sequences of 55 specimens (*Plasmodium*-positive, blood-fed and *Plasmodium*-negative) did not match reference sequences, possibly suggesting a previously unreported species, resolving as two clades.

**Conclusions:**

Our findings indicate the existence of yet-to-be identified and described anopheline species with a potential as malaria vectors in Kenya.

## Background

Entomological surveillance remains integral to effective intervention strategies towards malaria elimination. Previous studies have highlighted the importance of applying molecular approaches including sequencing in malaria entomologic surveillance, particularly involving species among known vector complexes as in the *Anopheles funestus* group [1–3]. This way, the identity and distribution of the species including their bionomic roles in malaria transmission can be accurately and comprehensively determined to guide appropriate measures in their control [3].

*Anopheles funestus* mosquitoes have been previously reported in semi-arid to arid areas of Kenya [4, 5]; however, these studies were not supported by molecular data. While *An. funestus* (*s.s.*) is the most ubiquitous species in the *An. funestus* group, our recent findings uncovered a higher number of sibling species including potential novel vectors not previously described in Kenya [3], including locality-specific occurrence. The findings are indicative of a poor knowledge of the full profile of sibling species in the group. Moreover, in many previous studies employing molecular methods to identify species within the group, a substantial proportion of specimens remain largely unidentified [3, 6, 7]. The aim of the present study was to explore the contribution of *An. funestus* mosquitoes to malaria parasite transmission in West Pokot County of Kenya, as part of disease monitoring. Malaria is prevalent in this semi-arid to arid ecology of Kenya [8], yet entomological knowledge of malaria vectors inhabiting such area remains poor.

## Methods

### Mosquito sampling

Adult females morphologically identified *An. funestus* (*sensu lato*) [9], collected as part of an arboviral disease monitoring project in Kacheliba (1°29'81"N, 35°01'80"E), West Pokot County (close to Uganda), were used in this study. The samples were collected outdoors in May 2016 using CO_2_-baited BG Sentinel traps (note: indoor collections using aspiration yielded none of this species). The weather in this area is hot and dry most of the year, with annual temperatures averaging about 21 °C. Rainfall is usually scarce and irregular from one year to the next with annual mean values ranging from about 300 to 400 mm. The mean daily rainfall during the sampling period in May 2016 was 5.55 mm. The main human activity in the area is nomadic pastoralism [5]. The common livestock in the area include cattle, goats and sheep.

### DNA extraction and identification of sibling species in the *Anopheles funestus* group

Genomic DNA extracted from individual whole specimens using Qiagen DNeasy Blood and Tissue Kit (Qiagen, Hilden, Germany) was used to identify by PCR the sibling species as well as probing for *Plasmodium* infection and host blood-meal sources. We identified species of the *An. funestus* group using an established cocktail of primers [10, 11], as previously described [3]. Briefly, PCRs were conducted on a SimpliAmp Thermal Cycler (Applied Biosystems, Loughborough, UK) in a 15 μl reaction volume containing 0.5 μM each of the primers, 3 μl of 5× Hot Firepol Blend Master Mix Ready to Load (Solis BioDyne, Tartu, Estonia) and 2 μl of DNA template. The cycling parameters were: initial denaturation at 95 °C for 15 min, followed by 30 cycles of denaturation at 95 °C for 30 s, annealing at 46 °C for 30 s and extension at 72 °C for 40 s, and a final extension at 72 °C for 10 min. Size fragments characteristic of each species were scored after separation in agarose gel electrophoresis (1.5%) stained with ethidium bromide against a 100 bp DNA ladder (O’ Gene Ruler, Fermentas, Fisher Scientific, Loughborough, UK).

### Detection of *Plasmodium* malaria parasites

Individual samples were tested for *Plasmodium* infection by analyzing high resolution melting (HRM) profiles generated from real time-PCR (RT-PCR) products of non-coding mitochondrial sequence (ncMS) [12] and/or amplification of the cytochrome *c* oxidase subunit 1 (*cox*1) gene [13] as previously described [3]. *Plasmodium falciparum* DNA obtained from National Institute for Biological Standards and Control (NIBSC; London, UK) was used as a positive control. Conventional PCR for *Plasmodium* spp. detection targeting the *cox*1 gene was carried out in a 15 μl reaction containing 0.5 μM of each primer, 9 μl of PCR water, 3 μl of 5× Hot Firepol® Blend Master Mix (Solis BioDyne) and 2 μl of DNA template. The amplifying conditions were: 95 °C for 15 min, followed by 40 cycles of 95 °C for 30 s, 59 °C for 30 s and 72 °C for 40 s, and a final extension at 72 °C for 10 min. PCR product of all amplicons were purified using ExoSAP-IT (USB Corporation, Cleveland, OH, USA) and outsourced for sequencing (Macrogen, Seoul, South Korea).

We further confirmed the species identity of *Plasmodium* sporozoite positive mosquito specimens by amplifying and sequencing of ribosomal DNA internal transcribed spacer region 2 (rDNA ITS2) [14] and/or the mitochondrial *cox*1 gene [15] as described previously [3]. We also amplified and sequenced 28 randomly selected specimens found negative for *Plasmodium* infection. The amplicons were purified as reported previously and outsourced for bidirectional sequencing (Inqaba Biotech, Pretoria, South Africa).

### Blood-meal analyses

PCR targeting the genes cytochrome *b* (*cytb*), *16S* ribosomal rRNA and *cox*1 were used to detect blood-meal host sources from the engorged specimen *Anopheles* by RT-PCR-HRM (Rotor Gene Q thermo cycler; Qiagen) and compared to profiles of known controls (positive: cow, Swiss mouse, pig, goat, chicken and human; negative: DNA from sugar-fed insectary-reared *Anopheles gambiae*) as described previously [3]. High resolution melting profiles generated were analyzed using HRM analysis tools present in the RGQ software (Qiagen). Vertebrate hosts were determined through comparison of the blood-meal HRM melt profiles to those of the standard reference control species.

### Sequence and statistical analyses

Mosquito and *Plasmodium* sequences were viewed and edited in Chromas, embedded in MEGA v.6 software [16] prior to querying the GenBank using BLASTn. Multiple sequence alignments of the resulting contiguous sequences (mosquito or *Plasmodium*) were performed using ClustalW in MEGA v.6 with default parameters. For mosquito sequences, maximum likelihood (ML) trees were constructed with nodal support for the different groupings evaluated through 1000 bootstrap replications utilizing the GTR+G and Jukes and Cantor model of sequence evolution for *cox*1 and ITS fragments, respectively. For ITS, indels were excluded from analysis. We further estimated the percent evolutionary divergence between the species found and reference species, including those of the *An. funestus* group, in MEGA v.6. The human blood index is expressed as the proportion of blood-feeding on humans of the total number of blood-fed mosquitoes examined.

## Results and discussion

We analyzed a total of 330 morphologically-identified *An. funestus* (*s.l*.) specimens, none of which amplified using the established ITS2 cocktail primers. *Plasmodium* infection was detected in two specimens. Analysis of the resultant 162 bp each of the sequenced isolates of the ncMS gene followed by Blastn searches in GenBank showed one of them as having 100% identity to *P. falciparum* (GenBank: CP017005) and the other with 100% identity to *P. ovale* (GenBank: AB354571).

**Fig. 1:**
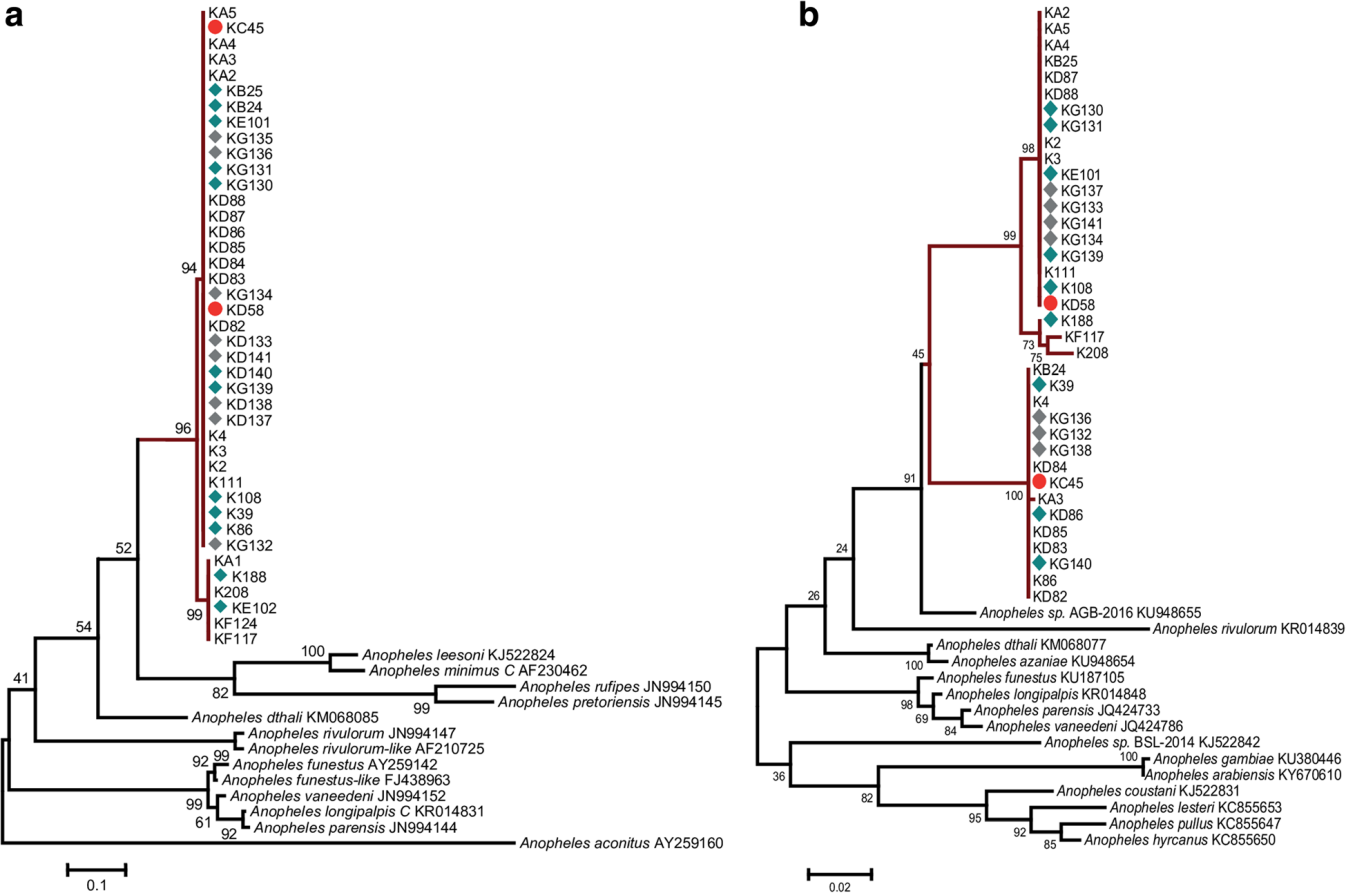
Maximum-likelihood tree inferred for mosquito specimens encountered in this study. **a** ITS2 sequences using a JC model (267–763 bp). **b** *cox*1 barcode region using a GTR+G model (615–658 bp). Analysis was performed in MEGA v.6 with bootstrap support values based on 1000 replications shown above nodes. Other reference anopheline species are included for comparison. The scale-bar indicates the number of substitutions per site. Study samples with *Plasmodium* infection are indicated with red block circles, and those with blood meals from humans or goats are highlighted in green and grey, respectively. Taxon abbreviations denote samples analysed with haplotype sequences corresponding to GenBank accession nos: **a** ITS2 (540–538 nt; MK043038-MK043040). **b** *cox*1 (528–646 nt; MK047664-MK047672)

Ribosomal ITS2 and mitochondrial *cox*1 sequences of the two PCR-*Plasmodium*-positive mosquito specimens could not be matched to reference anopheline sequences or known vector species in GenBank, BOLD or VectorBase databases (Fig. 1), suggesting the existence of a potentially important unreported malaria vector species.

Sequences of 25 blood-fed mosquito specimens (both ITS2 and *cox*1) were identical to those found *Plasmodium*-positive. Interestingly, a higher proportion of the blood-fed samples (21/25) had taken a blood meal from humans with an estimated human blood index of 84% for this previously unreported species. The remainder had fed on goats. Inclusion of sequences from selected *Plasmodium-*negative specimens in a phylogenetic tree, show that the species resolves as two well supported clades (Fig. 1) although with clade differences in the representation of the samples between the ITS and *cox*1 genes. The *Plasmodium*-positive specimens fell into a single clade for ITS and into each of the two clades for *cox*1 (Fig. 1). Select sequences of the mosquito specimens are available on GenBank: *cox*1 (528–646 nt; GenBank: MK047664-MK047672) and ITS2 (540–538 nt; GenBank: MK043038-MK043040). The species share 96% identity to the *Anopheles* sp. AGB-2016 (GenBank: KU948655), reported in Al’Sadah, Yemen. The next most closely related sequences (92% identity) are from other anopheline mosquito species (Fig. 1).

The bionomic traits uncovered for the species found in the present study, being trapped outdoors and with a high proportion feeding on humans, may compromise effective malaria control using the currently used indoor vector control tools, namely insecticide-treated bednets or indoor residual spraying. This species may contribute to stealth transmission and possibly sustenance of malaria in this focus. Our findings highlight the importance of employing molecular methods in entomological surveillance which target more than just the known malaria vectors. Furthermore, daytime activity is suggested for this species given that it was trapped using BG sentinel traps baited with CO_2_ set from 6:00 h to 18:00 h. A similar exhibition of such diurnal habit has recently been observed for *An. funestus* in West Africa; however, this was attributed to the scaling-up of universal coverage with long-lasting insecticidal nets [17]. Such a behaviour pattern could contribute to an increased risk of residual outdoor transmission, clearly representing a challenge for malaria control.

The specimens were morphologically identified as *An. funestus* (*s.l*.), although analysis *via* a molecular approach showed much divergence from sibling species in the group which are known to occur in Kenya. This finding, in part, highlights a taxonomic challenge in accurately identifying species morphologically [18]. Support against molecular evidence for mosquito species delineation has been highlighted [19] on account of sequence variation among individuals of the same species. However, molecular analysis has been highly instrumental in uncovering divergence among morphologically indistinguishable cryptic species [3, 20]. In fact, the DNA barcode targeting the *cox*1 gene has been widely used for mosquito species identification including discrimination for cryptic species groups, in conjunction with the ITS2 region [21]. Based on the barcode region, previous studies have suggested an evolutionary divergence of 2–3% as a threshold for intraspecific variation [22–25]. The average evolutionary divergence over sequence pairs between the putative species and any other species ranged from 0.5 to 15.1% for the *cox*1 sequence (Table 1). Observed levels of evolutionary divergence between this species and those in the Funestus group including other well-known malaria vectors in Kenya (*An. gambiae*, *An. arabiensis*, *An. coustani* [26]), surpasses this threshold, suggesting a separate genetic entity. Nonetheless, both morphological and molecular identification are useful for a detailed taxonomic elucidation of a given species important for vector surveillance and biodiversity studies [1, 27]. As such, further morphological descriptions and ecological studies of this species are warranted. It may also be important to link the molecular forms to iso-female lines that can be used to provide information on genetic variation within families. Our detection of *Plasmodium* infection in the newly-detected *Anopheles* species potentially implicates it in malaria transmission. However, whether the parasite would have been transmitted or not cannot be conclusively answered based on our analysis but its potential role as secondary vector in the more arid areas of Kenya should be further investigated. Confirming its role as a vector may benefit from additional studies to detect human *Plasmodium* sporozoites through dissection of the salivary glands.

**Table 1:** Estimates of average evolutionary divergence (%) over sequence pairs for the *cox*1 gene between the observed species and other reference species

Specimen	KC45	KD58	KF117	K208	K188	*An. rivulorum* KR014839	*An*. sp KU948655	*An. longipalpis* KR014848	*An. funestus* KU187105	*An. coustani* KJ522831	*An. gambiae* KU380446	*An. arabiensis* KY670610	*Anopheles* sp. KJ522842	*An. parensis* JQ424733
KC45														
KD58	5.1													
KF117	4.8	1.9												
K208	5.0	2.1	1.3											
K188	4.5	1.1	0.8	1.1										
*An. rivulorum* KR014839	9.3	9.5	9.5	9.5	9.0									
*Anopheles* sp. KU948655	2.6	3.7	3.7	4.0	3.4	9.0								
*An. longipalpis* KR014848	7.4	9.3	9.8	10.1	9.5	11.6	6.6							
*An. funestus* KU187105	6.9	8.7	9.3	9.5	9.0	10.6	6.6	1.1						
*An. coustani* KJ522831	9.8	9.8	10.6	10.8	10.3	11.4	8.7	7.9	8.5					
*An. gambiae* KU380446	13.2	14.0	14.8	15.1	14.3	11.6	12.4	10.1	10.1	8.5				
*An. arabiensis* KY670610	13.2	14.0	14.8	15.1	14.3	11.6	12.4	10.1	10.1	8.5	0			
*Anopheles* sp. KJ522842	10.1	10.3	10.6	11.1	10.3	10.1	9.8	9.8	9.3	9.5	10.0	10.1		
*An. parensis* JQ424733	7.9	9.3	9.8	10.1	9.5	10.6	7.1	1.1	1.6	6.9	9.0	9.0	8.7	
*An. vaneedeni* JQ424786	7.9	8.7	9.8	10.1	9.0	10.3	7.7	1.6	1.6	7.4	9.3	9.3	8.5	0.5

Taxon abbreviations follow those provided in Fig. 1 with GenBank accession nos MK047664-MK047672; *Anopheles* sp. KU948655 and *Anopheles* sp. KJ522842 represent an undescribed *Anopheles* species reported in in Al’Sadah, Yemen and western Kenya, respectively

*Anopheles funestus* has been known to have the widest distribution range among the Funestus group [28]. Its lack of detection including other previously reported species in the group from this drought-prone ecology is intriguing. Rainfall in this semi-arid to arid area of northern Kenya is often scarce and irregular from one year to the next [29]. Previous findings have suggested that *An. funestus* can inhabit extreme dry conditions in the Sahel depending on the availability of suitable breeding sites such as man-made irrigation zones [30, 31]. On the other hand, the influence of prolonged severe drought on population decline and elimination of *An. funestus* from parts of Africa has been reported [32], suggesting that extreme climate variability can affect the survival of this species. The close relatedness of the species encountered in this study to that from the Arabian Peninsula raises the possibility of adaptation of this and related species to very dry ecologies. While it may be inconclusive as to whether *An. funestus* and other known sibling species occur in Kacheliba given the limited duration of sampling, further studies over a longer period are encouraged to investigate the species composition and adaptation of mosquitoes in the Funestus group in this ecology.

## Conclusions

We have uncovered a species with potential as a malaria vector supported by sequence data and the exhibition of important bionomic traits such as the ability to feed on humans and that it was found infected with *Plasmodium* malaria parasites. The findings indicate the existence of yet-to-be identified and described anopheline species with potential as a malaria vector in the dry ecology of Kenya. More detailed studies including taxonomic description of the species and its ecological dynamics should be a focus of additional research.

## Abbreviations

CDC: Centers for Disease Control and Prevention
*cox*1: Cytochrome *c* oxidase subunit 1
HRM: High resolution melting
PCR: Polymerase chain reaction
*s.l.*: *Sensu lato*
*s.s.*: *Sensu stricto*
